# Bacteriophages as an Alternative Method for Control of Zoonotic and Foodborne Pathogens

**DOI:** 10.3390/v13122348

**Published:** 2021-11-23

**Authors:** Mohammed Mijbas Mohammed Alomari, Marta Dec, Renata Urban-Chmiel

**Affiliations:** 1 Faculty of Veterinary Medicine, University of Al Muthanna, Samawah 66001, Iraq; mijbas83@gmail.com; 2 Department of Veterinary Prevention and Avian Diseases, Faculty of Veterinary Medicine University of Life Sciences in Lublin, 20-033 Lublin, Poland; marta.dec@up.lublin.pl

**Keywords:** bacteriophages, foodborne pathogens, antimicrobial resistance, zoonotic bacteria

## Abstract

The global increase in multidrug-resistant infections caused by various pathogens has raised concerns in human and veterinary medicine. This has renewed interest in the development of alternative methods to antibiotics, including the use of bacteriophages for controlling bacterial infections. The aim of this review is to present potential uses of bacteriophages as an alternative to antibiotics in the control of bacterial infections caused by multidrug-resistant bacteria posing a risk to humans, with particular emphasis on foodborne and zoonotic pathogens. A varied therapeutic and immunomodulatory (activation or suppression) effect of bacteriophages on humoral and cellular immune response mechanisms has been demonstrated. The antibiotic resistance crisis caused by global antimicrobial resistance among bacteria creates a compelling need for alternative safe and selectively effective antibacterial agents. Bacteriophages have many properties indicating their potential suitability as therapeutic and/or prophylactic agents. In many cases, bacteriophages can also be used in food quality control against microorganisms such as *Salmonella*, *Escherichia coli*, *Listeria*, *Campylobacter* and others. Future research will provide potential alternative solutions using bacteriophages to treat infections caused by multidrug-resistant bacteria.

## 1. Introduction

Zoonotic pathogens cause problems all over the world, including diseases such as anthrax, brucellosis, bovine tuberculosis, hydatid disease, echinococcosis, trichinellosis, rabies, highly pathogenic avian influenza, Nipah/Hendra disease and bovine spongiform encephalopathy. In 2015, the WHO reported that more than 600 million people (1 in 10) worldwide fell ill as a result of foodborne infections [1,2]. According to the European Food Safety Authority (EFSA), the most frequently reported human foodborne diseases were campylobacteriosis and salmonellosis. However, the most dangerous pathogens for humans were identified as foodborne pathogenic bacteria found in livestock products, including enterohaemorrhagic *Escherichia coli* (EHEC; O157:H7), *Shigella* sp., *Enterococcus* spp. or *Listeria* spp. Multidrug-resistant pathogens isolated from human outbreaks, cattle, swine, and poultry were most often *S. aureus*, *Streptococcus* spp., *Vibrio* sp. and *Yersinia* spp. [3,4]. According to Niu et al. [5], these bacteria can also be transmitted to food products by direct contact with animals or indirectly by vectors such as insects, rodents, wild birds, or irrigation water.

The global increase in multidrug-resistant infections and antibiotic failures in control of pathogens has raised concerns in human and veterinary medicine. An official report of the European Food Safety Authority (EFSA) regarding zoonotic and indicator bacteria isolated from humans, animals, and food showed that a high proportion (28.6%) of human *Salmonella* strains were resistant to three or more antimicrobials, and 34.9% of *E. coli* strains isolated from pigs were resistant to more than six antibiotics [6].

There has been a marked increase in the antibiotic resistance of Gram-negative bacteria via a variety of mechanisms, such as antibiotic target modification, antibiotic degradation, and modulation of permeability through the bacterial membrane. These mechanisms have limited the development of novel antibiotics. The most resistant strains of bacteria are carbapenem-resistant *Enterobacteriaceae*, extensively drug-resistant (XDR) *Pseudomonas aeruginosa*, and XDR *Acinetobacter baumannii*. Understanding the mechanisms of resistance of multidrug-resistant bacteria is the main goal in the development of modern antibacterial agents [7].

Global livestock production is faced with an alarming increase in bacterial resistance, including among zoonotic pathogens. For example, Donkor et al., [8] showed higher antimicrobial resistance in livestock than in humans, with animal *E. coli* isolates exhibiting high levels of resistance to tetracycline and penicillin. This has led to renewed interest among scientists to develop alternative methods to antibiotics, including the use of bacteriophages, since the beginning of the 21st century [9].

Widespread multidrug resistance among bacteria necessitates the search for alternative methods of controlling infections, including pre- and probiotics, vaccines, bacteriophages, nanoparticles, antimicrobial peptides (AMPs) and others. An example is the use of bacteriophages to reduce or eliminate pathogenic bacteria in livestock production, as biocontrol agents to control foodborne pathogens and to reduce contamination on food-contact surfaces [9]. An important contribution to research on the use of bacteriophages to control bacteria, including zoonotic pathogens, is the development and implementation of new legal regulations in the EU regarding restrictions or complete bans on the use of selected groups of chemotherapeutics in individual sectors of animal production. An example of such legislative action is the EU Council Directive 2019/6 [10] coming into force in January 2022.

### 1.1. General Characteristics of Bacteriophages

Due to the widespread nature of bacteriophages (phages) associated with crops, live animals, and human intestinal environments, humans have direct and indirect contact with them. Many studies have demonstrated the common presence of bacteriophages in various fermented foods, such as yogurt and cheese. The application of specific bacteriophages to foods helps to reduce foodborne pathogenic bacteria [5].

Bacteriophages are bacterial viruses, causing complete lysis of a susceptible bacterial culture [11]. Interactions between phages and bacteria can be regarded as parasitism, as most virulent phage replication necessarily results in bacterial death. Certain interactions can be termed mutualistic, while some temperate phages encode benefits for the phenotypic properties of the host bacteria [12] According to Batinovic et al. [13], the prevalence of bacteriophages in the environment has been a natural phenomenon for billions of years, resulting in a balance of commensal and pathogenic bacteria. Phages and bacteria are the oldest and most ubiquitous microorganisms on Earth, likely having originated approximately 3 billion years ago [14,15].

Phages are prevalent in a variety of environments, including water, forest groundcover, food products, wastewater, and animal and human waste [16]. Bacteriophages have also been detected in commercial products, such as sera and human vaccines, as well as inside the human mouth (dental plaque and saliva) and in the gastrointestinal tracts of animals and humans [17].

Although bacteriophages may be present autonomously outside the host, all phages require the bacterial cell as a host for multiplication. Most phages are highly specific for host cell surface receptors such as receptor binding proteins (RBPs) or LPS [18,19].

### 1.2. History of Bacteriophages

Bacteriophages were first discovered more than 100 years ago by two microbiologists, Frederick Twort from England and the French Canadian Felix d’Herelle [20,21]. The first experimental and successful phage therapy was carried out by D’Herelle in the control of fowl typhoid in chickens (95–100% survival) [22]. He also coined the term ‘bacteriophage’, meaning ‘bacteria eater’. Finally, in 1940, electron microscopes were used to identify the viral nature and morphology of phages [23]. Bacteriophages have been used in various types of therapies in humans, e.g., in dermatological, ophthalmological, urological, paediatric, otolaryngology and surgical infections. The significant therapeutic success of these treatments had a major impact on the development of phage therapy in the pre-antibiotic era. This was crucial, as the only treatment available in the first two decades of the 20th century was serum therapy (e.g., for pneumococci or the diphtheria bacterium), so bacteriophage therapy began to dominate in human medicine [24].

The discovery of the antimicrobial properties of *Penicillium notatum* in 1928 by Alexander Fleming culminated in the successful development of the first major antibiotic, penicillin, in 1941 [25], which marked the beginning of the antibiotic era and naturally inhibited the development of bacteriophage therapy.

At present, as bacterial resistance to antibiotics is increasing significantly worldwide, phages are one of the factors with potential to replace them [26]. The best known bacteriophage centres in the world are the Eliava Institute of Bacteriophages, Microbiology, and Virology (EIBMV) of the Georgian Academy of Sciences, in Tbilisi, Georgia, and the Hirszfeld Institute of Immunology and Experimental Therapy (HIIET) of the Polish Academy of Sciences, in Wroclaw, Poland. Both institutes offer phage therapy against many bacterial and fungal pathogens, e.g., *Staphylococcus* spp., *Klebsiella* sp., *Proteus* sp., *E. coli*, and *Pseudomonas* sp., as well as other enteric pathogens [27,28,29].

### 1.3. Classification of Bacteriophages

Bacteriophages are the most widespread life forms on Earth. By 2018 year more than 650 strains of bacteriophages had been deposited in the American Type Culture Collection (ATCC) and >27,000 bacteriophage nucleotide sequences had been deposited in the International Nucleotide Sequence Database Collaboration (INSDC) [30]. The total number of these bacterial viruses has been estimated at 10^32^, which is 10 times the number of characterized bacteria. In water, the total count of bacteriophages has been estimated at 10^4^ to 10^8^ virions/mL^−1^ [31].

**Figure 1:**
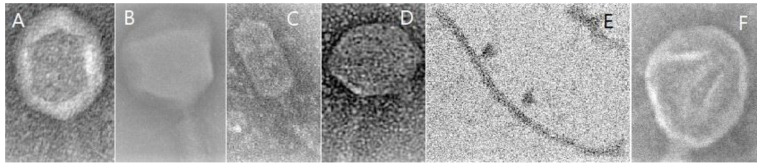
Examples of capsid structures in bacteriophages in TEM microscopy. (**A**) Helical; (**B**) Polyhedral; (**C**) Prolate; (**D**) Icosahedral; (**E**) Filamentous; (**F**) Pleomorphic-like.

The classification of bacteriophages is based on the type of nucleic acid (ssRNA, dsRNA, ssDNA, dsDNA), the structure of the capsid (e.g., helical, pleomorphic, icosahedral, filamentous/thread-like, complex/polyhedral), which is built of structural proteins, and their life cycle, bacterial target, and site (Figure 1). The phage taxonomy criteria applied by the International Committee on Taxonomy of Viruses (ICTV) were nucleic acid composition and virion morphology [9]. In 2015 the Bacterial and Archaeal Viruses Subcommittee (BAVS) classified phages into 873 species, 204 genera and 14 subfamilies [32]. The classification of bacteriophages is still ongoing, and in 2018 the ICTV presented a new classification of these bacterial viruses into 142 families, 81 subfamilies and 4978 species [9]. Most bacteriophages (96%) belong to the order *Caudovirales*, which is grouped into three main families: *Myoviridae*, *Podoviridae* and *Siphoviridae* [32]. Most bacteriophages contain double-stranded DNA, and the nucleic acid is coated with a protein capsid. Some phages have an additional layer (envelope) [17]. As new bacteriophages are continually being detected, their classification is constantly modified. The latest classification of viruses, based on the virus taxonomy proposed by the ICTV, was presented in our previous paper [33].

### 1.4. Life Cycles of Phages

The life cycle of phages is an important element of infections of bacteria. Phages can be categorized into types based on their virulence: lytic (virulent, productive) and lysogenic (temperate, dormant). Virulent phages follow a lytic cycle in the bacterial cell and lyse it to release a newly created population of phages [34].

The lytic cycle includes the adsorption, penetration, biosynthesis, assembly and release of bacteriophages from the infected bacterium. During this process many phages use specific proteins located on the surface of the bacterial cell as receptors. During the adsorption phase, the bacteriophage adheres to the bacterial cell, and phage proteins bind to specific receptors, such as teichoic and lipoteichoic acid for Gram-positive bacteria or LPS for Gram-negative bacteria [35]. The next phase, penetration, consists of destruction of the bacterial wall by bacteriophage enzymes and insertion of the genetic material into the bacterial cell. This is followed by the formation of capsid structures for nucleic acid and protein replication, accompanied by inhibition of replication of bacterial DNA. The phage genetic material is transcribed in the bacterial cell by RNA polymerases to produce mRNA, which supresses host intracellular synthesis as a consequence of bacteriophage multiplication [36]. Tens, hundreds or thousands of replicated phages are released by means of lysis of the bacterial cytoplasmic membrane by a phage protein (holin) and the formation of pores by endolysin encoded by double-stranded phage DNA and peptidoglycan hydrolases. The duration of the entire lytic cycle may be 20–40 min or up to 1–2 h [9,37].

Lysogenic infection via phages involves integration of their genetic material into the chromosome of the infected bacteria (prophage), which does not destroy the bacterial cell or produce a new population of bacteriophages. It leads to the integration of the phage genetic material with the bacterial DNA and its transmission into a new population of bacteria. This kind of bacteriophage is called a temperate phage, and in cells carrying a prophage it is referred to as lysogenic. Nevertheless, the viral prophage, also called an endogenous phage (a latent form of phage), can become activated by abnormal environmental conditions and other external stress factors that can damage the bacterial genetic material, including sunlight, UV radiation, some alkylating cytostatics (chlorambucil, cyclophosphamide, ifosfamide, estramustine or chlormethine) or mutagenic antibiotics such as mitomycin C. In some cases, the prophage is excised incorrectly from the chromosome, taking with it neighbouring bacterial genes. This is one of the main means of horizontal gene transfer (HGT) among bacteria, which is also one of the main methods in molecular biology [9]. Phages which have been defined as temporary include *E. coli* Lambda [38], with activity against *E. coli* and other *Enterobacteriaceae*; phage Mu, specific for *Salmonella*, *Citrobacter* and *Erwinia*; MM1 *Streptococcus pneumoniae*; and φ11 *S. aureus* [39].

In another type of life cycle involving chronic infection, bacteriophages infect the bacterial cell, in which new phage populations arise without destroying the bacteria. The chronic infection lifestyle is found in rod-shaped (filamentous) single-stranded DNA phages and in plasmaviruses that infect mycoplasmas. In the chronic infection lifestyle, phages are gradually eliminated from the bacteria over a long period without destruction of the cell [40].

## 2. The Spectrum of Use of Bacteriophages

The specificity of phage activity means that they infect only the bacteria specific for them (called the host) via external receptors, which determines the phage host range. Therefore, the use of phage therapy relies on a detailed and accurate characterization of the bacteria, including pathotypes and serotypes. Bacteriophages can be used in a variety of forms and methods to control and eliminate bacteria, including therapy, food protection and sanitation procedures [1,9]. Examples of the scope of the use of bacteriophages are presented in Table 1.

Bacteriophages can potentially be used as biological control agents, especially in the reduction and elimination of bacterial contamination in foods, e.g., by *Salmonella*, *Listeria monocytogenes*, *Campylobacter* spp. or *E. coli* O157:H7 [15,50]. The high efficacy and safety of bacteriophage therapy is due in part to their specificity for selected bacteria: a single species, serotype, or strain. This is beneficial because the commensal gut microbiota is not destroyed. Another advantage is that, due to the self-replication of bacteriophages at the site of application, repetition of the application is often unnecessary. In many cases, no side effects of clinical treatment are observed, indicating a high level of safety that has been confirmed in many studies [51]. However, the application of bacteriophages in live animals or humans induces a cellular immune response, which could lead to the inactivation of phages, rendering them ineffective in eliminating bacteria [52,53,54].

In many experimental phage therapies a beneficial effect was observed as a significant reduction in bacterial content or elimination of the pathogens. Phages have been used to control Shiga-toxin-producing *E. coli* (ETEC) infections in newborn ruminants, including calves and lambs, or other livestock species, such as piglets [41,55]. They have been exploited to control bacterial infections in humans in many countries, including Poland, Georgia, Russia, France, Belgium, Switzerland and the USA [56,57,58]. Phage therapies have been applied against infections caused by numerous pathogens, especially multidrug-resistant bacteria, e.g., *Acinetobacter*, *Burkholderia*, *Citrobacter*, *Enterobacter*, *Enterococcus*, *Escherichia coli*, *Klebsiella*, *Morganella*, *Proteus*, *Pseudomonas*, *Shigella* spp., *Shigella flexneri*, *Staphylococcus*, *Salmonella*, *Serratia* and *Stenotrophomonas*. According to the Eliava Phage Therapy Centre, bacteriophage therapy against *Enterococcus faecalis*, *E. coli* (O11, O18, O20, O25, O26, O44, O55, O113, O125 and O128), *Proteus vulgaris*, *Proteus mirabilis*, *Pseudomonas aeruginosa*, *Salmonella* spp., and *Shigella* spp. showed positive results in 35–50% of human patients [59,60].

### Bacteriophage Interactions during Phage Therapy

Bacteriophages are regarded as the most applicable ecological and alternative means of elimination of pathogens (control and prevention of infections) due to their natural origin and numerous advantages, including the following:

- lysis of bacteria usually highly resistant to antibiotic therapy, living in a biofilm;
- high degree of safety for commensal and symbiotic flora;
- possibility of use with other bacteriophages as a cocktail or with other antibacterials;
- complete biodegradability of bacteriophages, making them safe for the organism and the environment [17].

However, phage therapy may carry a risk of immunological reactions, which is linked to the protein structures of bacteriophages. The immune response to bacteriophages depends on the location of the bacterial infection and the route of administration of the phages. The activity of bacteriophages also relies on their ability to penetrate epithelial cells and potentially spread to the bloodstream, lymph and internal organs such as the lung, liver, kidney and brain [61]. Bacteriophages can activate dendritic cells to synthesize pro-inflammatory factors (including IL-6, IL-1α, IL-1β and TNF-α) and to induce changes in the expression profile of these cell surface proteins and activation of the NF-κB signalling pathway [62].

The results of many studies confirm that bacteriophages can be phagocytosed by mammalian cells [63]. For example, Geier et al. [64] demonstrated rapid removal of wild-type phage λ from the circulatory system in humans. According to the authors, phagocytosis via immune cells is the main process of elimination of bacteriophages in mammals, and this mechanism takes place during lysis of bacteria by bacteriophages, which increases the activity of phagocytic cells, including PMN cells. The higher number of neutrophils at the site of infection is necessary to remove phage-resistant bacteria; this neutrophil-phage cooperation process has been confirmed in the resolution of *P. aeruginosa* infections [53,65]. However, some studies [66] have confirmed that bacteriophages can also express anti-inflammatory properties by decreasing the expression of TNFα and monocyte chemoattractant protein-1, which reduces ROS production by neutrophils and protects the epithelia against damage.

Some bacteriophages can also be a natural component of the intestinal microbiota and consumed food [67]. The oral administration of phages against *Staphylococcus*, *Klebsiella*, *Escherichia*, *Proteus* and *Pseudomonas* also induces the production of antibodies [68]. There has been no evidence of immunological disorders following phage ingestion *per os* at any concentration [69]. Topical application of phages to animals and humans also caused no side effects [70].

Minor problems have been observed in the case of other internal organs and blood vessels, which are not natural environments for phages. Here the immunogenic and immunomodulatory effects of phages can be observed. Bacteriophages can have non-specific effects on the immunological functions of various immune cells, including PMNCs, as well as on cytokine production and the induction of specific antibodies against non-phage antigens [71]. For example, resident liver macrophages (also called Kupffer cells) are able to eliminate bacteriophages by phagocytosis four times faster than spleen macrophages. The natural innate immune response is usually sufficient to eliminate pathogens before the activation of adaptive immune mechanisms. Bacteriophages can activate immune mechanisms and thus affect the metabolic activity of immune cells. However, bacteriophages can inhibit the production and release of reactive oxygen species in response to pathogens, which could decrease innate antibacterial immunity. [72]. Phages can induce antibodies that neutralize them, which can inhibit the antibacterial effect of phages in the form of lysis of targeted bacteria [71,73]. It is not currently clear how long this type of antibody will remain in the body, as knowledge of the kinetic aspect of bacteriophage activity is insufficient. Moreover, the titre of these antibodies depends on many factors, including the route of application (local application causes a minor increase in antibodies) and its frequency [74]. Some information about the influence of bacteriophages on immune responses in animal’s model has been presented in Table 2.

Antiphage antibodies are probably one of the most important factors influencing the efficacy of phage therapy. However, the activation of the production of neutralizing antibodies by phages need not be a problem during the initial phase of treatment of bacterial infections, because bacteriophage activity is much faster than the production of phage-neutralizing antibodies [27]. However, these antibodies can affect the efficacy of treatment during the second phase of therapy. This necessitates the implementation of additional solutions, such as the following:

- repeating phage administration two or more times, because bacteriophages can multiply at the site of application during infection of the host bacteria;
- increasing the phage concentration in the solution, because a high level of phages protects against complete destruction by neutralizing antibodies;
- using different phages, especially during the second and subsequent cycles of application during treatment, because resistance differs from one phage to another [27]. In addition to the increase in neutralizing antibodies during phage therapies, the concentration of class M and G immunoglobulins increases as well and continues to increase with subsequent applications of phage preparations [83,84].

Besides activating humoral response mechanisms, phages also play a significant role in the modulation of cellular immunity against them. For example, s.c. application of MS-2 phages induced a hypersensitivity reaction in guinea pigs [85]. It has been suggested that the cellular response plays only a minor role in phage inactivation, as observed in the case of phage T7 in T cell-deficient mice [79]. In another study [86], phages had an immunosuppressive effect by suppressing the activation of T lymphocytes during the development of transplantation tolerance.

While bacteriophage therapies have been an effective tool in control of bacterial infections in various animal species, phages are also currently used for typing and diagnosis of specific bacterial species and for control of foodborne pathogens in food.

## 3. Commercial Products with Bacteriophages for Elimination of Foodborne Zoonotic Pathogens

Foodborne infections are the most important global health problem, contributing significantly to hospitalizations and deaths worldwide despite many advances in pathogen surveillance. Traditional food sanitation techniques using antimicrobial methods (including pasteurization, high pressure, irradiation or chemical treatment) can reduce pathogens in foods in varying degrees. However, these methods may damage equipment and adversely affect the organoleptic qualities (and possibly the nutritional value) of foods. The most important problem with using chemicals is that they eliminate ‘good’ microbes, which are beneficial in natural preservation of foods [87]. Therefore, it seems preferable to use an effective natural and ecological alternative such as bacteriophages for biocontrol of foodborne pathogens. Bacteriophages are mainly used in three sectors of the food industry to ensure food safety: primary production, biopreservation and biosanitization. As components of commercial products, they are currently finding application in the elimination of pathogens from food products of animal origin (meat products, milk and dairy products) or plant origin (fresh fruits and vegetables).

The number of commercial bacteriophage products approved for use in food safety in various countries is continually increasing. Many commercial companies around the world have shown interest in information about the use of bacteriophages as antibacterial tools to control foodborne pathogens, e.g., in the United States (AmpliPhi Biosciences (VI, USA), Enbiotix (Boston, MA, USA); Intralytix), the United Kingdom (Novolytics, Sarum Biosciences and Fixed Phage, Bolton, UK), EU countries such as France (Pherecydes Pharma, Romainville, Ile-de-France, France) and Portugal (Technophage and InnoPhage, Lisbon, Portugal), and other countries [88]. Detailed information about commercial bacteriophage products used for biocontrol of foodborne pathogens in food is presented in Table 3.

## 4. Advantages and Disadvantages of Bacteriophage Therapy

Phages have several advantages over antibiotics as therapeutic agents, such as activity against all types of bacteria, including MDR-pathogens. Their narrow antibacterial spectrum (which protects the natural microbiome), the low level of side effects, and their extensive distribution when administered systemically are also worth noting. They also may exert an effect on the inflammatory response, and their low production cost and high efficacy are significant benefits [31,99]. Many studies have confirmed the beneficial effects of the use of bacteriophages, shown as follows:

Bacteriophages show high specificity for their target pathogens and kill only pathogens without destroying the physiological saprophytic flora; the narrow host range of phages is also a useful feature in prophylaxis of infections caused by enteric bacteria [100].

The distribution of phages in the body following systemic administration is much more extensive than in the case of antibiotics, in part due to the lack of or very low level of resistance of bacteria [31].

- Because the mechanism of action of phages against the host bacteria is different to that of antibiotics, they are highly effective against many pathogens, especially against multidrug-resistant bacteria [36].
- Phages replicate at the site of infection even after a single application, because they multiply inside the bacterial cell and therefore are released at the site of infection [101].
- Bacteriophages are resistant to stress factors during food production [91].
- Phage therapy is theoretically cheaper than antibiotic therapy due to the simplicity of production [99]. The unit costs of production as well as the costs of isolation and characterization are comparable or even lower than the costs of classical chemotherapeutic products [102].
- There is no withdrawal period in livestock due to the lack of residue in tissues as soon as therapy is completed [103].
- There are no side effects or allergic reactions because most bacteriophages consist mainly of proteins and genetic material (DNA or RNA).

However, in addition to the positive effects of phage therapies, widespread use of bacteriophages is limited by obstacles such as the following:

- Due to their high specificity for a single type of bacteria, bacteriophages have a narrow host range [104].
- Bacteriophages may neutralize antibodies, which may prevent a portion of the administered phage dose from adhering to the target bacteria [104].
- Bacteriophages have poor stability in the environment, e.g., sunlight, UV, low pH <3.5, or high temperature >50°C [17,105].
- Only lytic phages are admissible in phage therapy because lysogenic (temporary) phages may be a source of horizontal transfer of bacterial toxins or antibiotic resistance [31].
- The duration of survival of phages is varied, depending in part on the presence of the host bacteria. Their activity is also influenced by the environment within the organism in which it is administered, and therefore the survival of phages must be monitored at the site of administration in order to assess their antimicrobial activity [99].
- Information about the kinetics of phages remains insufficient, especially the degree of adsorption, the number of replications necessary for a therapeutic effect, the latent period, and their elimination from the body by phagocytic cells [106].

## 5. Bacteriophage Efficacy in Experimental Models

There are many methods of application of phages in therapies for humans and animals, including intraperitoneal, subcutaneous or intramuscular injection or oral, intragastric, rectal, topical or intranasal administration. Forms of administration of phages during therapy include sprays, aerosols, lozenges, compresses, mouthwash, suppositories, throat rinses, bandages, eye or ear drops and tampons [107]. In many studies in humans and animals, the form of application and type of administration has been associated with the type and location of the disease. In earlier studies [108,109,110,111], the best therapeutic effect was observed after direct application of phages to the target bacteria, as in the case of bacterial dysentery caused by *Shigella*; intestinal dysbacteriosis caused by *E. coli* and *Proteus* spp.; lung and pleural infections caused by *Staphylococcus*; suppurative skin infections caused by *Pseudomonas*, *Staphylococcus*, *Klebsiella*, *Proteus*, and *E. coli*; and infections of the skin or nasal mucosa caused by *Klebsiella* spp.

Some studies have explored the use of phages for control and treatment of neonatal enterotoxigenic *E. coli* infections in cattle, poultry and pigs [40,112]. Bacteriophages have also been used in controlling systemic infections with foodborne pathogens, including *Salmonella* spp., *E. coli*, *Campylobacter* spp., *Vibro* spp., *Pseudomonas aeruginosa*, and other pathogens, such as *Staphylococcus* spp., *Streptococcus* spp., *Klebsiella* spp., *Acinetobacter* spp., and even *Mycobacterium* spp. These experiments were carried out in experimental mouse or rat models, as well as in chickens, rabbits, calves, pigs and sheep. Examples of the effects of experimental phage therapies in different animal species and in the control of various pathogens are presented in Table 4.

## 6. Conclusions

To sum up, bacteriophages have many properties indicating their potential suitability as therapeutic or/and prophylactic agents. Future research on the scope of phages will provide a good picture of their potential to treat infections caused by multidrug-resistant bacteria. However, as bacteriophages are essentially ‘living’ drugs, the study of their use for therapy or biocontrol spans from purely clinical observations to molecular analysis to considerations of immunology and ecology. Due to the antibiotic resistance crisis, there is a compelling need for alternative safe and selectively effective antibacterial agents.

## Figures and Tables

**Table 1:** Examples of the use of bacteriophages in controlling bacteria.

Scope of Use	Example	Host Pathogens	References
Treatment of human and animals	Gastroenteric, respiratory, urinary tract and skin infections, otitis, keratitis	*E. coli*, *Salmonella* spp., *S. aureus*, *Pseudomonas* spp., *Enterococcus* spp., *Acinetobacter baumanni*	[1]
Prophylaxis and treatment	Neonatal diarrhoeal infections in calves;	*E. coli*,	[41]
	*Campylobacter* infections in broiler chickens;	*Campylobacter jejuni*	[42]
	*Salmonella* infections in chickens	*Salmonella* spp.	[43]
Decontaminants	Biocontrol agents against food- and beverage-borne pathogens	Control of LAB growth during ethanol fermentation	[44,45]
Biosanitization	On equipment surfaces to eradicate biofilms in food production; on plastic, glass, and ceramic surfaces in hospitals	*S. aureus*, *E. coli*, *P. aeruginosa*, *L. monocytogenes Acinetobacter baumannii*	[46,47]
Bio-preservation	Highly processed products with a short shelf life	*Listeria monocytogenes*; *Campylobacter* spp.	[48]
Agriculture	Biocontrol of plant pathogens, i.e., potato and tomato diseases; onion scab; lettuce and leek diseases; fruit tree diseases; cultivated mushrooms	*Pseudomonas* spp., *Xanthomonas* spp.*, Erwinia* spp., *Ralstonia* spp., *Agrobacterium* spp., *Xylella* spp., *Pectobacterium* spp., *Dickeya* spp. *Pleurotus ostreatus*	[49]
Aquaculture	Biocontrol of fish pathogens in commercial fish farming	*Mainly to Vibrio* spp., less to *Edwardsiella* spp., *Lactococcus* spp., *Pseudomonas* spp., *Aeromonas* spp., *Flavobacterium* spp.	[49]

**Table 2:** Examples of the influence of bacteriophages on immune responses in animals.

Kind of Phage	Form of Application	Animal Model	Influence on Immune Parameters	References
*Pseudomonas* spp. bacteriophage (PA1Ø)	100 µL of PA1Ø (5 × 10^4^ PFU; 5 × 10^7^ PFU (10 MOI) or 5 × 10^8^ PFU (100 MOI) in a single i.p. dose	4–5-week-old male ICR mice weighing 24–26 g	Increase in phagocytosis (killing effect of PA1Ø + PMN up to 6 h after application)	[65]
*Pseudomonas aeruginosa* phage PAK_P1	Intranasally at a curative dose of 1.0 × 10^8^ PFU or 1.0 × 10^9^ PFU	Wild-type BALB/c (C), wild-type C57Bl6/J (B6)	Increase in neutrophil activity, NK cells; reduced production of IFNγ and TNFα	[75]
*Cronobacter sakazakii* ES2 phage	Phage suspension 10^6^ PFU·mL^−1^ in vitro	6–8-week-old C57BL/6 mice	Increase in expression of maturation markers CD86, CD40, and MHC II; stimulation of induction of NF-κBp65-mediated-IL-12p40; stimulation of IL-12 expression; suppression of IL-6, TNF-α, IL-1β, and IFN-γ	[62]
*E. coli* T4 phage	Intraperitoneal injection at 20 µg/mouse	Female C57Bl6/J (6–8-week-old) mice	No effect on production of cytokines IL-1α, IL-6, IL-12, and TNF-α; minor changes in expression of MHC II, CD40, CD86, and CD80	[76]
*E.coli* T4 bacteriophages	T4 phages 5 × 10^8^ PFU/mL	8–12-week-old female C57BL/6 mice	Inhibition of specific antibody response; reduction in bacteria-induced ROS production by phagocytic cells; antitumour response; activation of T cells for IFN-γ production	[77]
*E. coli* P1 and P2 phages	10^6^ PFU/mL in vitro	Mice	Stimulation of TNFα; stimulation of macrophage activity in vitro	[78]
Wild-type *E. coli* T7 phage	10^9^ PFU/mL injected in vitro into tail vein of mice	Adult female C57BL/6J, SCID (C57BL/6J-Prkdcscid), B-cell-deficient (C57BL/10-Igh-6tm1Cgn) and T-cell deficient (C57BL/6J-Hfh11nu) mice	Spontaneous antibodies, mainly IgM, observed in sera; slight effect on NK activation; anti-inflammatory effect—ROS suppression	[79]
Wild-type *E.coli* φ26, φ27, φ29	10^7^–10^8^ PFU/mL for 5 days per rectum as suppositories	25 newborn HF calves aged 1 d to 2 weeks	Significant increase in IgG and IgA production stimulation of nonspecific immune response—IFNγ, lysozyme; activation of acute phase response SAA and HP	[41]
Wild-type *E.coli* phage and bacteriophage genomes NC-A: MK310182; NC-B: MK310183; NC-G: MK310184	3 × 10^7^ PFU/mL of phage mixture with drinking water	8-week-old germ-free Swiss Webster mice	Whole bacteriophages and phage DNA stimulated IFN-γ via nucleotide-sensing receptor TLR9	[80]
*S. aureus* vB_SauM_JS25 phage	MAC-T cells pre-treated with vB_SauM_JS25 phage 10^8^ PFU/well for 3 h	In vitro MAC-T bovine mammary epithelial cells	Reduction in TNF-α, IL-1β, IL-6, IL-8, and IL-10	[54]
*Staphylococcus* spp. bacteriophage A3R or 676Z	3 doses of 10^10^ PFU/mouse in drinking water and peritoneally	C57BL/6J normal male mice	Induction of specific antibodies in blood (IgM, IgG, IgA)	[81]
*Klebsiella pneumoniae* MTCC109 bacteriophage PA43	Intranasal application of 10^9^ PFU BPA43 phage after 2 h of bacterial infection	BALB/c mice, 6–8 weeks old, weight 20–25 g	Suppression of local inflammatory reaction in lungs; suppression of migration of lymphocytes and macrophages	[82]

**Table 3:** Examples of commercial bacteriophage products used in biocontrol of foodborne pathogens in various foods.

Commercial Phage Product	Target Bacteria	Company	Target Food Products	Country Approving Product	References
SalmoLyse^®^	*Salmonella* spp.	Intralytix, Inc., USA	Raw pet food ingredients; meat products: chicken, tuna, turkey; plant products: cantaloupe, lettuce	USA	[89]
SalmoFreshTM	*Salmonella* spp.	Intralytix Inc., USA	Poultry, fish and shellfish, fresh and processed fruits and vegetables	USA, Canada, Israel	[90]
PhageGuard S SalmonelexTM	*Salmonella* spp.	Micreos Food Safety/Nederlands	Fresh poultry meat	USA, Canada, Australia, Israel	[91]
Bafasal^®^	*Salmonella* spp., *Aeromonas* spp. *Pseudomonas* spp., *Yersinia* spp.	Proteon Pharmaceuticals (Łódź, Poland)	Regulatory-approved poultry feed	Poland	[9]
EnkoPhagum	*Salmonella* spp., *Shigella* spp.; enteropathogenic *E. coli*; *Staphylococcus* spp.	Brimrose Technology Corporation (Sparks Glencoe, MD, USA)	Meat products	Georgia	[92]
BacWash TM	*Salmonella* spp.	OmniLytics Inc. (Sandy, UT, USA)	For disinfection of skin of live animals prior to slaughter	USA	[4]
Biotector^®^ S	*Salmonella* Gallinarum *S.* Pullorum	CJ CheilJedang Research Institute of Biotechnology (Seoul, Korea)	In animal feed to control *Salmonella* in poultry	South Korea	[93]
PhageGuard STM	*Salmonella*	Micreos Food Safety BV (Wageningen, The Netherlands)	Fresh poultry meat	Netherlands, Australia, Canada, USA	[87,94]
EcoShield TM	*Escherichia coli* O157:H7	Intralytix Inc. (Columbia, MD, USA)	Kosher meat (ground beef); vegetables (tomatoes, broccoli, spinach); lettuce and cantaloupe; leafy greens	USA	[9,91,95]
Secure Shield E1	*Escherichia coli* O157:H7	FINK TEC GmbH (Hamm, Germany)	Beef carcasses	USA	[96]
EcoShield PX™	*Stx Escherichia coli* O157:H7	Intralytix, Inc., Baltimore, MD, USA	Fresh-cut leafy greens; foods of plant origin, beef, chicken	USA, Canada, Israel	[90,95]
ShigaShield™ (ShigActive™)	*Shigella* spp.	Intralytix, Inc., Baltimore, MD, USA	Beef, poultry, dairy products, including cheese; fruit and vegetable surfaces	USA	[90,97]
ListShield™	*Listeria monocytogenes*	Intralytix, Inc., Baltimore, MD, USA	Food biopreservative in meat and poultry products	USA, Canada, Israel	[9,87,90]
Listex P100 PhageGuard Listex™	*Listeria monocytogenes*	Micreos Food Safety, Wageningen, Netherlands	Beef and turkey meat; fish and shellfish; dairy products; red smear soft cheese, smoked salmon and fresh salmon; frozen vegetables	USA, Australia, New Zealand, Israel, Switzerland, the Netherlands	[87,98]
ListPhage™	*Listeria monocytogenes*	Intralytix, Inc., Baltimore, MD, USA	Pet food	USA, EU	[91]
Agriphage™	*Xanthomonas campestris* pv. *vesicatoria*, *Pseudomonas syringae* pv. *tomato*	OmniLytics Inc., USA	Foods of plant origin, especially tomatoes and peppers	USA	[91]
Agriphage-Fire Blight	*Erwinia amylovora*	OmniLytics Inc., USA	Surfaces of apples and pears	USA	[91]
Biolyse™	*Erwinia*, *Pectobacterium*, *Pseudomonas*	APS Biocontrol Ltd./Dundee, UK	Vegetables, including potatoes	UK, Europe	[91]

**Table 4:** Examples of major experimental studies on phage therapy in animals.

Animal Species	Pathogen Species	Phage Treatment	Results	Treatment Procedure	References
Cattle–newborn Holstein-Friesian heifers	*E. coli* O9:K30.99 10^6^ CFU mL^−1^	Oral administration of phage cocktail (B44/1 and B44/2), 10^11^ PFU mL^−1^	100 % reduction of mortality in calves; Significant reduction (93%) of morbidity of bacterial diarrhoea; high protection against ETEC infections	Treatment of diarrhoea	[113]
Cattle–Holstein-Friesian dairy cows	*Staphylococcus* *aureus*	Direct infusions into teats with bacteriophage K cocktail (CS1, DW2) (10^8^ PFU ml^−1^)	About 10,000-fold reduction of *S. aureus* in udder; lower presence of somatic cells in milk	Treatment of subclinical mastitis	[114]
20 female BALB/cJRj (SPF) mice	*Staphylococcus aureus* causing mastitis in cows	Inoculation with 10^8^ PFU of ISP phage mixture into mammary glands	Significant reduction of bacterial count; reduction or lack of clinical changes in mammary glands	Antibacterial activity and therapeutic effect	[115]
280 Holstein-Friesian lactating cows with metritis during the first and second lactations	*Escherichia coli* strains causing metritis	Intravaginal administration of 20 mL 10-phage cocktail 10^9^ PFU mL^−1^ at 230, 260 and 275 days of gestation	Lack of antibacterial effect; no prophylactic effect in prevention of metritis; increased incidence of retained placenta	Failure of therapeutic and prophylactic effect in metritis	[116]
25 newborn Holstein-Friesian heifers aged 0–14 days old	*E. coli* causing diarrhoea in newborn calves	Rectal application as suppositories of phage cocktail (26, 27, 29 at 10^7^ to 10^9^ PFU mL^−1^) mixed with *Lactobacillus* spp. strains for 5 days	Significant reduction of clinical signs and duration of diarrhoea <24h; significant reduction of ETEC content in faeces 2 log_10_ CFU/mL; protection against re-infection for 3 weeks after treatment; immunomodulatory effect	Prophylactic and therapeutic effect against diarrhoea	[41]
Holstein-Friesian dairy cows with clinical or subclinical mastitis	*S. aureus* strains obtained from cows with subclinical and clinical mastitis, pig farm and human infections	0.1 mL phage cocktail (STA1.ST29, EB1.ST11, and 27) 1.2 × 10^8^ PFU/mL or 1.2 × 10^9^ PFU/mL against *S. aureus* inoculated into about 5.0 mL of milk obtained from cows with mastitis	Significant reduction of *S. aureus* in milk–2 log_10_ CFU/mL in vitro	Antibacterial activity	[117]
3 female Yorkshire pigs weighing~60 kg	*S. aureus* ulcers	*S. aureus* F44/10 and F125/10, inoculated topically at 10^8^ to 10^9^ PFU	Slight reduction of *S. aureus* strains, reduction of ulcerous changes	Therapeutic effect on skin ulcers	[118]
16 small pigs 3 to 4 weeks old	*Salmonella enterica* ser. Typhimurium at 5 × 10^8^ CFU mL	Microencapsulated alginate beads containing 16-phage cocktail (*SEP-1*, *SGP-1*, *STP-1*, *SS3eP-1*, *STP-2*, *SChP-1*, *SAP-1*, *SAP-2*), ∼10^9^ to 10^10^ PFU/mL by gavage	Significant early reduction (99%) in concentration of *S*. Typhimurium 2 to 3 log_10_ CFU/g in the ileum, caecum and tonsils; significant influence on health status and AWG of pigs	Prophylactic and therapeutic effect	[119]
3-week-old weaned pigs	*E. coli (ETEC); O149:H10:F4*	Oral administration of phage cocktail GJ1–GJ7 or mono-phage: prophylactic 10^10^ PFU/pig or therapeutic 10^8^ PFU/pig	Significant reduction of diarrhoea; reduction of duration of diarrhoea <2 days, mean diarrhoea score, and mean composite diarrhoea score significant reduction of ETEC strains; protection against diarrhoea	Prophylactic and therapeutic effect against diarrhoea	[120]
Weaned pigs >4 weeks old	Oral challenge with 5 mL of 109 CFU/mL *Salmonella* Typhimurium	Microencapsulated phage cocktail in feed (5 × 10^11^ PFU) for 5 days before challenge with *Salmonella* Typhimurium	Reduction of *S.* Typhimurium in ileum and caecum by about 1 log_10_ CFU/g	Therapeutic and prophylactic effect	[121]
4-week-old weaned pigs	*Salmonella enterica* serovar Typhimurium	5 mL of a 8- phage cocktail at 10^9^ PFU/mL (SEP-1, SGP-1, STP-1, SS3eP-1, STP-2, SChP-1, SAP-1, SAP-2)	Significant reduction of *Salmonella* Typhimurium; 100% lytic activity against 34 *Salmonella* reference strains and 92.5% lytic activity against 107 wild strains	Therapeutic effect in diarrhoea	[122]
Merino cross wethers sheep (1 year of age)	*S. aureus* strain ATCC 25923	Phage cocktail CTSA 2 × 10^8^ PFU/mL applied to right and left sinuses	Reduction of tissue damage; reduction of *S. aureus* colonization	Therapeutic and antibacterial activity	[123]
20 Canadian Arcott rams weighing 50 kg	*E. coli O157:H7*(10^9^ CFU/mL	Oral administration of *E. coli* phage cocktail P5, P8 and P11 (10^10^ PFU) administered orally 5 times using a sterile 60-mL syringe and stomach tube	Significant reduction~2 log_10_ CFU of intestinal *E. coli* O157:H7 in sheep; total elimination of bacteria in 30% of animals	Prophylactic and therapeutic effect	[124]
Ross broiler chickens at 34 d of age	*S. enterica* ser. Enteritidis P125109; *S. enterica* serotype Typhimurium 4/74; *S. enterica* serotype Hadar 18	Bacteriophage suspensions as antacid administered by oral gavage 9.0 or 11.0 log_10_ PFU of φ151 (*S. enterica* ser. Enteritidis), φ25 (*S. enterica* ser. Hadar), or φ10 (*S. enterica* ser. Typhimurium)	Significant reduction of *S. enterica* ser. Enteritidis and Typhimurium caecal colonization by ≥4.2 log_10_ CFU within 24 h	Therapeutic and prophylactic effect	[125]
Young chicks	*Salmonella* Typhimurium DT104	Single oral dose of phage FO1 of 10^9^ (PFU)/chick in encapsulated form	Reduction of *Salmonella* Typhimurium strains in caecum	Antibacterial effect	[126]
Vrolix chicks aged 20 days	*Campylobacter jejuni*	3-bacteriophage cocktail 5 × 10^8^ PFU of CP14, CP81 or CP68	Reduction of *C. jejuni* strains in caeca by approx. 3 log_10_ CFU units	Antibacterial and protective effect	[127]
Chickens	*Campylobacter jejuni*; *S. enterica* serovar Enteritidis	Direct inoculation onto chicken skin, *C. jejuni* typing phage 12673 at 10^6^ PFU/cm^2^ of skin; *S. enterica* serovar Enteritidis phage P22, phage 29C, 10^3^ PFU/cm^2^ of skin	Significant reduction of *Campylobacter* up to 2 log_10_ per unit area of skin within 48 h; reduction of *C. jejuni* ~2 log_10_ on experimentally contaminated chicken skin after phage application	Therapeutic and antibacterial effect	[128]
Ross strain 308 commercial chicken broilers	*Salmonella enterica*	3-phage cocktail, liposome/alginate, encapsulated, 10^10^ PFU/animal for 9 days	Significant decrease in *Salmonella* spp. concentration (~50%) in caeca	Antibacterial activity	[129]
Broiler chickens (Cobb 500) at 1 d of age	*E. coli* ser 02	Sprayed with 200 mL of 8 × 10^8^ PFU/mL phage SPR02	Significant reduction of mortality by >10%	Antibacterial and protective effect	[130]
8-day-old quail	Oral challenge with 100 μL of 1.2 × 10^9^ CFU ml^−1^ *S.* Enteritidis	Oral application of 100 μL of 10^6^ PFU ml^−1^ bacteriophage for 3 days	Reduction of *S.* Enteritidis in caecal tonsils of Japanese quails to 33.3 and 20%, 24 h and 7 days after application; prophylactic effect against *S.* Enteritidis colonization, increase in resistance against *Salmonella* challenge	Prophylactic effect	[43]
2-day-old New Zealand White rabbits	Oral infection with *Vibrio cholerae* 8 × 10^8^ CFU	Oral application of 3 phages (Phi_2, 24 and X29) 10^9^ PFU	Reduction of bacteria count up to 4 log_10_ CFU/g; full protection against clinical signs of disease	Prophylactic and therapeutic effects	[131]
120 eight-week-old female BALB/c mice	*Mycobacterium* *ulcerans* as ulcerous infections	Single dose of mycobacteriophage D29 10^8^ PFU/mouse administered 33 days post infection	Progressive reduction of footpad swelling by day 150 post-infection significant reduction of *M. ulcerans*~1.5–2 log_10_ CFU/ml	Therapeutic effect and antipathogenic activity effect	[132]
Mice	*Pseudomonas aeruginosa*	Bacteriophage PAK_P1 intranasally at curative dose of 1.0 × 10^8^ PFU/mL or prophylactic dose of 1.0 × 10^9^ PFU (MOI 100)	Prophylaxis of acute respiratory infections caused by *P. aeruginosa*; significant reduction of clinical signs; resistance to infection; stimulation of immune response	Therapeutic and prophylactic effect	[75]
BALB-C female mice aged 10 weeks	*Pseudomonas aeruginosa*	Single dose of phage MMI-Ps1 10^7^ PFU suspension by intranasal application	Prophylaxis against *P. aeruginosa* infection; significant reduction of bacterial content in lungs about 2 log_10_	Protective and antibacterial effect	[133]
Female mice C57BL/6 mice, aged 7 to 8 weeks	*Acinetobacter baumanni*	*A. baumanni* phage Bϕ-C62 inoculated intranasally (1 × 10^10^ PFU/ml	100% survival after challenge with *A. baumanni*	Therapeutic effect, slight immunostimulatory effect	[134]
BALB/c mice aged 6–8 weeks	*Klebsiella pneumonia*-induced pneumonia	Bacteriophage suspension 2 × 10^9^ PFU/mouse applied in a single dose i.n.	Significant decrease in duration of illness and microscopic lesions; suppression of necrosis, bronchiolitis, and infiltration of inflammatory cells	Therapeutic effect	[82]
BALB/c mice	*Klebsiella pneumoniae* B5055	50 μL of 10^8^ PFU/mL single and 5-phage cocktail applied topically at wound site (Kpn1, Kpn2, Kpn3, Kpn4 and Kpn5)	Significant reduction of *K. pneumoniae* load to 4.32, 4.64, 4.42, 4.11 and 4.27 log CFU/mL; rapid healing of wounds in all phage-treated groups	Therapeutic and antibacterial activity	[135]
Male Wistar rats; 9–10 weeks old	*Staphylococcus aureus*-associated pneumonia	Intravenous application of cocktail of 4 phages (2–3 × 10^9^ PFU/mL of 2003, 2002, 3A, and K	Increase in survival from 0% to 58% significant reduction of bacterial content in the lung (1.2 × 10^6^ CFU/g of tissue for survivors vs. 1.2 × 10^9^ CFU/g for nonsurviving animals); reduction of lung damage	Therapeutic and immunomodulatory effect; antibacterial activity	[136]
New Zealand White infant rabbits (aged 3 days) and CD-1 infant mice (aged 4 and 5 days)	*Vibrio cholerae*; oral administration of 5 × 10^8^ CFU/rabbit or mouse	Oral administration of phage cocktail (3 × 10^7^ or 10^8^ PFU/rabbit or mouse)	Protective effect against cholera via significant reduction of caecal colonization by *V. cholerae*; protection against cholera-like diarrhoea	Prophylactic and therapeutic effect	[137]
New Zealand White rabbits 2-day-old	*Vibrio cholera* 5 × 10^8^ CFU per animal	Phage Phi_1 at 1 × 10^9^ PFU/animal orally administered either 6 h before or 6 h after bacterial challenge	Protection against clinical signs of cholera; lack of diarrhoea; significant reduction of 2–4 log_10_ CFU/g *V. cholera*	Prophylactic and therapeutic effect	[131]
Female C57BL6/SJL mice as cow mastitis infection model	*Streptococcus dysgalactiae* NRRL B-65273, *S. agalactiae* NRRL B-65272, and *S. uberis* NRRL B-65274	Direct application into mammary gland: *Streptococcus* spp. phage endolysins 25 μg/gland for λSA2, 250 μg/gland for B30, and 12.5 (λSA2) + 125 (B30) μg/gland	Significant reduction of *S. dysgalactiae* content by 3.5 log_10_ CFU; *S. agalactiae* (2 log); *S. uberis* (4 log); protection against clinical signs of mastitis	Therapeutic effect and antibacterial activity	[138]

## Abbreviations

AMPs	antimicrobial peptides
ATCC	American Type Culture Collection
BALB	Bagg Albino Mouse
BAVS	Bacterial and Archaeal Viruses Subcommittee
CD	cluster of differentiation
CFU	colony-forming unit
EFSA	European Food Safety Authority
EHEC	Enterohaemorrhagic Escherichia coli
ETEC	Enterotoxigenic Escherichia coli
EFSA	European Food Safety Authority
EIBMV	Eliava Institute of Bacteriophages, Microbiology, and Virology
HF	Holstein–Friesian
HIIET	Hirszfeld Institute of Immunology and Experimental Therapy
HGT	horizontal gene transfer
Hp	haptoglobin
ICR	Institute of Cancer Research
ICTV	International Committee on Taxonomy of Viruses
IFNγ	Interferon gamma
Ig	immunoglobulin
i.p	intraperitoneally
i.n	intranasal
INSDC	International Nucleotide Sequence Database Collaboration
IL	interleukin
LAB	lactic acid bacteria
LPS	lipopolysaccharide
MAC-T	mammary alveolar cells
MDR	multidrug-resistant
MHC	major histocompatibility complex
MOI	multiplicity of infection
NF-κB	nuclear factor kappa-light-chain-enhancer of activated B cells
NK	Natural killer
PFU	plaque-forming units
PMN	polymorphonuclear
RBPs	receptor-binding proteins
ROS	reactive oxygen species
mRNA	messenger RNA
SAA	serum amyloid A
ssRNA	single-stranded ribonucleic acid
dsRNA	double-stranded ribonucleic acid
ssDNA	single-stranded deoxyribonucleic acid
dsDNA	double-stranded deoxyribonucleic acid
SPF	specific free pathogens
stx	Shiga toxin
TEM	transmission electron microscopy
TNF-α	tumour necrosis factor α
UV	ultraviolet
WHO	World Health Organization
